# In Pursuit of Delay-Related Brain Activity for Anticipatory Eye Movements

**DOI:** 10.1371/journal.pone.0073326

**Published:** 2013-09-09

**Authors:** Melanie R. Burke, Graham R. Barnes

**Affiliations:** 1 Institute of Psychological Sciences, University of Leeds, Leeds, West Yorkshire, United Kingdom; 2 Faculty of Life Sciences, University of Manchester, Manchester, Lancashire, United Kingdom; University Medical Center Groningen UMCG, Netherlands

## Abstract

How the brain stores motion information and subsequently uses it to follow a moving target is largely unknown. This is mainly due to previous fMRI studies using paradigms in which the eye movements cannot be segregated from the storage of this motion information. To avoid this problem we used a novel paradigm designed in our lab in which we interlaced a delay (2, 4 or 6 seconds) between the 1^st^ and 2^nd^ presentation of a moving stimulus. Using this design we could examine brain activity during a delay period using fMRI and have subsequently found a number of brain areas that reveal sustained activity during predictive pursuit. These areas include, the V5 complex and superior parietal lobe. This study provides new evidence for the network involved in the storage of visual information to generate early motor responses in pursuit.

## Introduction

The ability of the brain to anticipate future motion is essential for survival as without this ability we would find the simple task of crossing the road or driving a car extremely hazardous. Anticipation in pursuit eye movements describes the ability to generate smooth eye movements prior to the availability of retinal motion information, in the expectation of future target motion; it thereby overcomes the inherent neural lag in visual motion processing. This anticipation uses past information to predict future trajectories and hence, to a large extent, anticipation of future motion requires storage of motion information from prior exposure [1]. However, the areas of the brain responsible for this storage process have not been clearly identified. Ocular pursuit provides a good example in which to examine predictive motor control because of the wealth of available behavioural and neurophysiological data. The brain areas involved in smooth pursuit eye movements are now relatively well understood and include early visual areas, V5, frontal eye fields (FEF), supplementary eye fields (SEF), and other intra-parietal and frontal regions (for review see [2]). The role of early visual areas is predominantly in visual processing with V5 playing a substantive role in motion processing (velocity information) [3], [4], the frontal eye fields play an important role in initiating the motor command via the superior colliculus (SC) [5], and corrective saccades during pursuit [6]. The supplementary eye fields are more involved in rule encoding [7], and preparation in the decision to pursue [8], [9]. Finally, the intra-parietal regions play a role in the multimodal integration and coordinate transformations required to convert sensory information into a motor output [10]. It is now well established that two parallel pathways are involved in initiating and maintaining pursuit via the cerebellum: 1) a direct pathway from MT/MST to the Dorsolateral Pontine Nucleus (DLPN), and 2) a more indirect one from MT/MST via FEF to the Nucleus Reticularis Tegmenti Pontis (NRTP) (for review see [11]). Furthermore, the MT/MST to DLPN pathway appears to be more sensitive to retinal image velocity and eye velocity, whereas the FEF to NRTP pathway is more sensitive to eye acceleration. The two pathways may thus play separate roles in initiating and maintaining pursuit (for review see [12]).

Attempts to identify sites specifically associated with anticipation in pursuit have isolated a number of brain areas, including visual area 5 (V5), the frontal eye fields (FEF), the supplementary eye fields (SEF), the dorsolateral prefrontal cortex (DLPFC) and anterior cingulate (ACC) [13]–[16]. Unfortunately, these areas are also involved to some extent in non-predictive pursuit and no studies to date have been able to segregate areas specifically associated with anticipation from areas associated with generating the motor response. In the present study we have used a novel paradigm that integrates a Go (Active)/NoGo (Passive) task with a predictive smooth pursuit tracking task in an attempt to isolate the brain mechanisms involved in motion storage for anticipation from those involved in generating the eye motor response. The rationale for the method is as follows.

The initiation of pursuit is normally dependent on visual motion processing, which incorporates large delays (∼80 ms). To overcome such delays the pursuit system is able to generate anticipatory movements that can be revealed if there is a strong expectation that target motion will occur in the future in association, for example, with a timing cue [17], [18]. The ability to scale the velocity of such anticipatory eye movements is dependent on prior exposure to target motion information, suggesting that velocity information is retained in some form of memory. The ability to initiate an appropriately scaled anticipatory response after an intervening fixation period, which may last as long as 14 s [19], indicates that this velocity information can be stored for prolonged periods. Moreover, Poliakoff, Collins, & Barnes [20] have shown that active following of a target is not necessary for creation of the stored information; passive observation is sufficient, although a further study by Burke & Barnes [21] revealed that the level of anticipatory pursuit was slightly degraded after passive viewing using the task reported here. A previous paper by Burke and Barnes [22] has evaluated fMRI data associated with this task by performing a block design comparing the NoGo and Go tasks and reporting results from across the whole trial. This previous paper succeeded in revealing the brain areas involved when inhibiting a pursuit eye movement. The objective of the present study was to identify areas of the brain that are crucial for the storage of motion information for anticipatory pursuit; an event-related design was used to examine brain activity during the delay periods between the initial presentation of a moving target (acquisition phase) and a subsequent attempt to pursue that target in a second presentation (response phase). In this way we have been able to examine storage-related activity in the absence of motor output and visual motion input. In addition, by instructing subjects to either actively pursue (Active task) or passively observe (Passive task) the moving target in the initial presentation, we have been able to identify whether there is modified activation in storage-related areas or possible involvement of additional areas in the Active task to account for this motor advantage observed in behaviour [21].

## Materials and Methods

Please note the following behavioural method has been described previously [21].

### Subject Population

This study was approved by the North West Research Ethics Committee for Greater Manchester North for the study titled: “Development of short-term memory for motion in the brain during active and passive viewing of a visual stimulus” (REC reference: 07/Q1405/32). In-line with this ethical agreement informed written consent was obtained from each of the eleven healthy volunteers that took part in the study of which five were male (mean age: 29.7 years; standard deviation: 8.5 years). All subjects had no neurological or visual defects with good visual acuity. Two subjects wore contact lenses throughout the experiments in both the laboratory and scanning environments.

### Tasks

Subjects were given verbal and written instructions prior to performing the set of tasks listed below, and provided with information sheets and consent forms to complete. All 11 subjects performed the equivalent eye movement tasks in a laboratory session approximately 1 week prior to the fMRI scanning session. Each subject performed 3 blocks in each of these experimental sessions, consisting of 48 pairs of presentations in each block (resulting in 144 pairs in total) lasting 50 minutes. The design for the presentation of the stimulus has been reported previously [22]. The 144 paired tasks (Active, Passive, Random and Control) were randomized within each block, creating an event-related design. This randomization was used to minimise the predictable effects between trials within a block and therefore isolate the predictive effects to within the paired trial. The stimulus was presented in pairs in either a predictable or randomized manner, in which either the first and second presentations were matched in time and velocity, or the two presentations were randomized in both time and velocity, respectively. The velocity of the target was randomized between pairs for the predictable task but remained constant within the pair, whereas velocity was randomized both between and within the pairs for the random task. The fixation cue was a white square that subtended ∼1 dva on the eye that either changed colour or remained white to indicate which of the tasks the subjects would subsequently perform (Green  =  Active, Magenta  =  Passive, green and black  =  Random, and white  =  control). The target again subtended ∼1 dva on the eye and was a coloured disk (green or magenta depending on task) that moved up, down, left or right at either 15°/s or 30°/s. All experiments took place in a darkened scanning room to minimise any external light source un-related to the task. The following 4 tasks (Active, Passive, Random and Control) were presented in random order in, but in equal numbers within each block (resulting in 12 repetitions of each task within each block).

#### Active task

**Figure 1:**
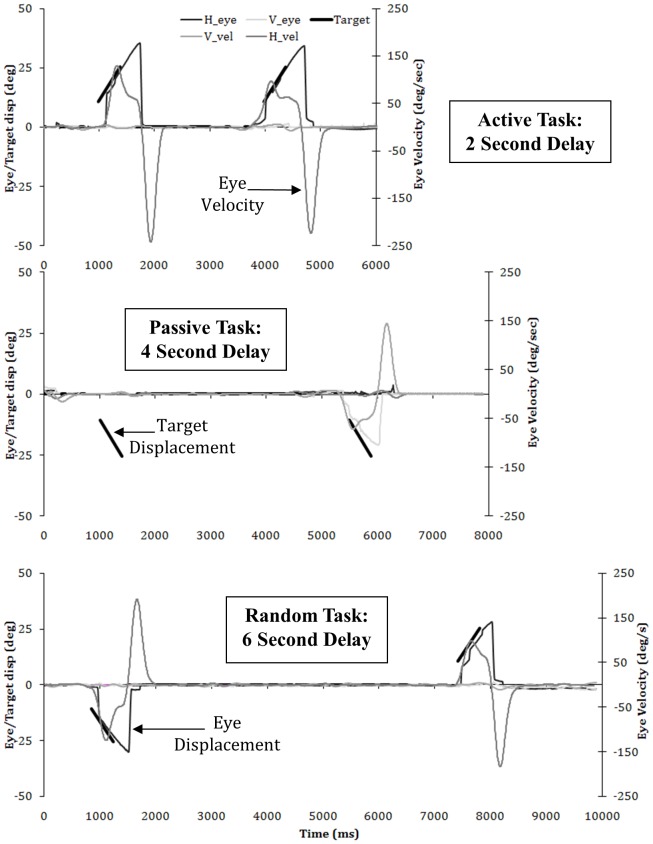
A typical individual subject's eye movement responses (displacement and velocity) from the lab to each of the 3 main tasks displayed with 3 difference delays (i) Active task with 2 second delay (upper graph), (ii) Passive Task with 4 second delay (middle graph) and (iii) Random Task with 6 second delay (lower graph). Displacement of the eye uses the left-hand scale and is shown by either the darkest (horizontal) or lightest (vertical) grey. Velocity is shown by the mid-greys (horizontal  =  darker and vertical  =  lighter) with the velocity scale depicted on the right-hand side of the graphs.

This task consisted of a white fixation cue visible for 200 ms that subsequently changed colour to green for a further 200 ms before the screen went blank (gap) for 400 ms. After the gap the green cue and a green target (T1) appeared, with the target displaced towards the direction of motion (3° or 6°) and smoothly moving at either 15°/sec or 30°/sec for 800 ms before being extinguished. A randomized delay of either 2, 4 or 6 seconds was then included, in which only the fixation cue was visible before the same cue and target presentation was repeated as above. The subjects were informed that they must follow the green moving target when it appeared and fixate the centrally positioned cues (Figure 1, upper graph).

#### Passive task

This task is similar the task described above however, the white fixation cue changed to magenta instead of green in the first presentation indicating the subject must maintain fixation while a target would smoothly move in their peripheral field of view. Again a 2, 4 or 6 second delay was used which was then followed by the cue turning green indicating the subject to follow the preceding target in the second presentation (as above) (see Figure 1, middle graph).

#### Random task

This task was designed to mimic the designs above and was cued with a green square with a black cross inside, however this time the duration of the gap was randomized (200–600 ms) and also the direction and speed (i.e. velocity) of the target between each of the two presentations in the pair. This randomization ensured the subjects could not predict the timing or direction of the target. The subjects were instructed to simply follow the green target when it appeared (see Figure 1, lower graph).

#### Control task

This task was designed to mimic the timing of the stimuli in the above tasks but did not involve the subject moving their eyes. A white square target appeared in the centre of the screen for 400 ms after which the target disappeared during a randomized gap (200–600 ms). The target then reappeared in the centre of the screen for 800 ms before a blank screen was again presented for 2, 4 or 6 seconds. This presentation was then repeated. Like the previous tasks all the various tasks were balanced.

### Equipment set-up and acquisition

**Figure 2:**
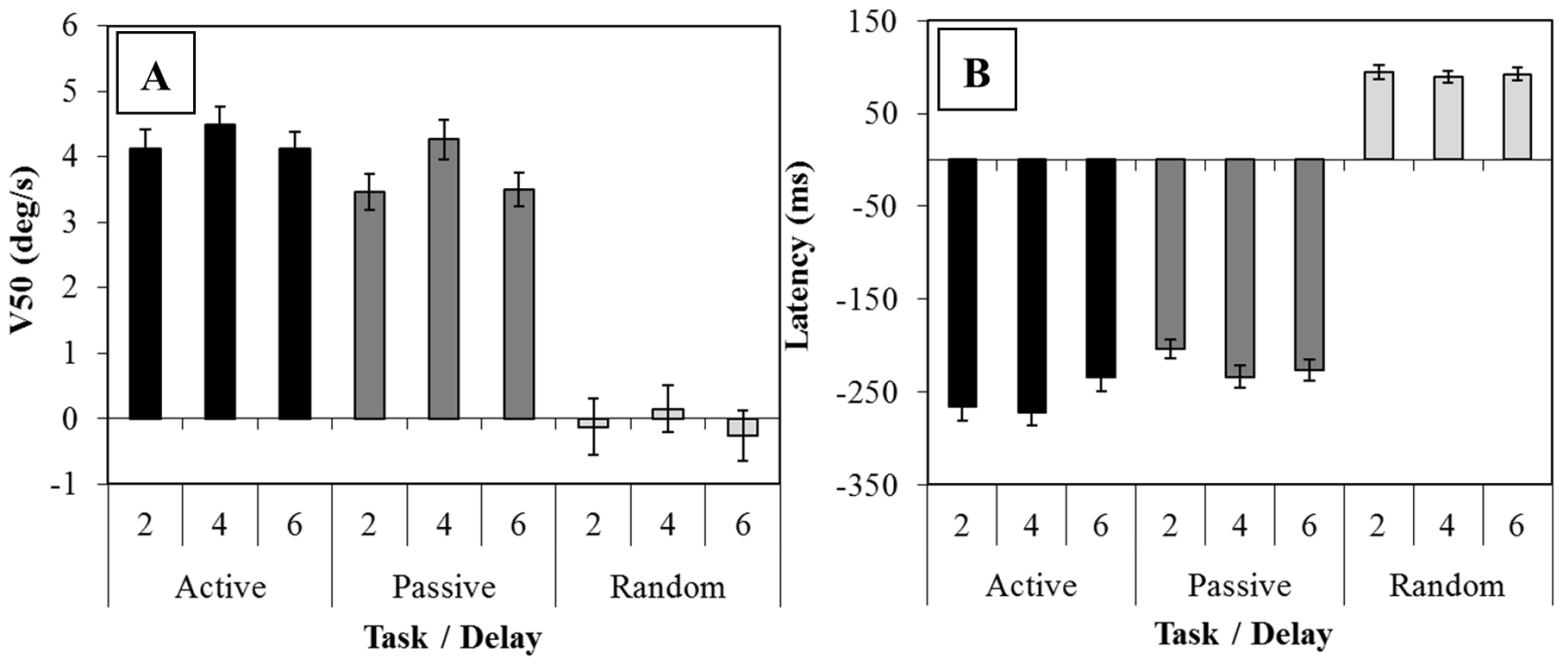
The mean (± std) eye velocity 50 ms after target onset (V50) (i.e. prior to visual feedback) (graph A), and mean (± std) latency of the eye movement from target onset (graph B) for all subjects is shown. Data is displayed according to task (Active, Passive and Random) and delay (2, 4 and 6 seconds).

Eye movements were monitored to the above task in both a laboratory setting using the Chronos eye tracker running at 200 Hz (Chronos Vision GmbH, Germany) [21], and inside the fMRI scanner using the ASL long range optics video eye tracker (Applied Science Laboratory (ASL), USA) running at 60 Hz. When subjects lay supine in the scanner, an image of the right eye was reflected into the ASL video camera positioned near the head of the subject via a mirror positioned on the head coil. The visual stimuli was generated using COGENT software (http://www.vislab.ucl.ac.uk/cogent) running in a MatLab (Mathworks Inc., MA, USA) environment. This system was linked to a liquid crystal projector, which back-projected the image onto a large white screen situated at the feet of the subject. The subject was able to see the stimulus via a mirror positioned on the headcoil. Head movements were minimized during the task by the use of foam padding either side of the head. The eye movements were analysed offline by capturing the pupil from the video image. Many of the resultant eye movement data files from the scanner proved noisy and difficult to interpret. However, qualitative comparisons of the scanner and laboratory data with additional visual inspection of a video image of the eye during the scanning provided evidence that subjects performance was equivalent in both laboratory and scanner environments. The quantitative results reported here (figure 2) are therefore taken from the higher resolution eye-tracker in the laboratory environment.

We used functional magnetic imaging at 3T (Philips 3.0T Achieva) with an 8 channel SENSE head coil (Achieva 3.0T Neuro Coil) specially designed for greater signal to noise ratio.

### Data Analysis

Details of the eye movement analysis for the laboratory based experiment and fMRI experiment have been described in detail previously [21], [22] (please see Figure 1). This eye movement data formed a 3×3 ANOVA and the results of the main effects across the whole trial (pair) for the Active and Passive tasks for the 11 subjects used in the current study, have been reported separately [22].

Initially a T_1_- weighted axial image for each subject was obtained. We then measured blood-oxygenation level-dependent (BOLD) changes in cortical activity during the tasks. During each scan we implemented a T2* sensitive echo planar imaging pulse sequence with a repetition time (TR) of 2000 ms, an echo time (TE) of 30 ms and flip angle of 90°. Each volume comprised 30 slices of the full brain at using 3×3×3 mm^3^ voxel size and a field of view of 256 mm.

We applied standard pre-processing procedures to the resultant fMRI data using SPM2 (http//www.fil.ion.ucl.ac.uk/spm/) that included; slice time correction, spatial realignment, normalization to MNI coordinates and a 9 mm full-width half maximum Gaussian filter. The data were high passed filtered (128 s cutoff) and global drifts were removed with proportional scaling. A design matrix was created which modelled each task and delay separately (Tasks: Active (a), Passive (p), Random (c) and Control; Delays: 2 s, 4 s, 6 s). To avoid any “double dipping effects” (Kriegeskorte et al, 2011) we generated ROI's based on coordinates of activity from a previous study looking at anticipation in pursuit eye movements [15]. The selected ROI's were also confirmed in a more recent study using the same paradigm [22]. The current study differs from the previous study [22] in that only activity during the delay is reported. To achieve this we extracted the delay within each trial by firstly splitting each of the 9 conditions into 3 event components: (1) ***Acquisition phase*** (1^st^ presentation of stimulus), (2) ***Delay phase*** and (3) ***Response phase*** (2^nd^ presentation of stimulus). This resulted in 27 conditions in total (1a2, 2a2, 3a2, 1a4, 2a4, 3a4, 1a6, 2a6, 3a6, 1p2, 2p2, 3p2, 1p4, 2p4, 3p4, 1p6, 2p6, 3p6, 1r2, 2r2, 3r2, 1r4, 2r4, 3r4, 1r6, 2r6, 3r6). The control task was also modelled in the same manner for removal of stimulus related activity in the tasks of interest (Active, Passive and Random). The delay phase was modelled on the last 2 seconds of each delay period (e.g. between 2 and 4 seconds in the 4 second delay) so all delay (memory) components were equal in time. This delay data for each subject formed the basis for the first level “fixed effects” (FFX) GLM analysis and had little in common with activity observed across the whole trial. This analysis specifically addressed the activity during a delay in pursuit that allowed interrogation of the signal in the absence of the eye movement and stimulus. From this individual data a one-sample t-test for each condition was generated for all subjects thus providing a group level “random effects” (RFX) analysis. Activated brain areas were identified using SPM2 anatomy toolbox (v1.6, Eickhoff et al, 2007) using MNI coordinates.

### Region of Interest Analysis

**Figure 3:**
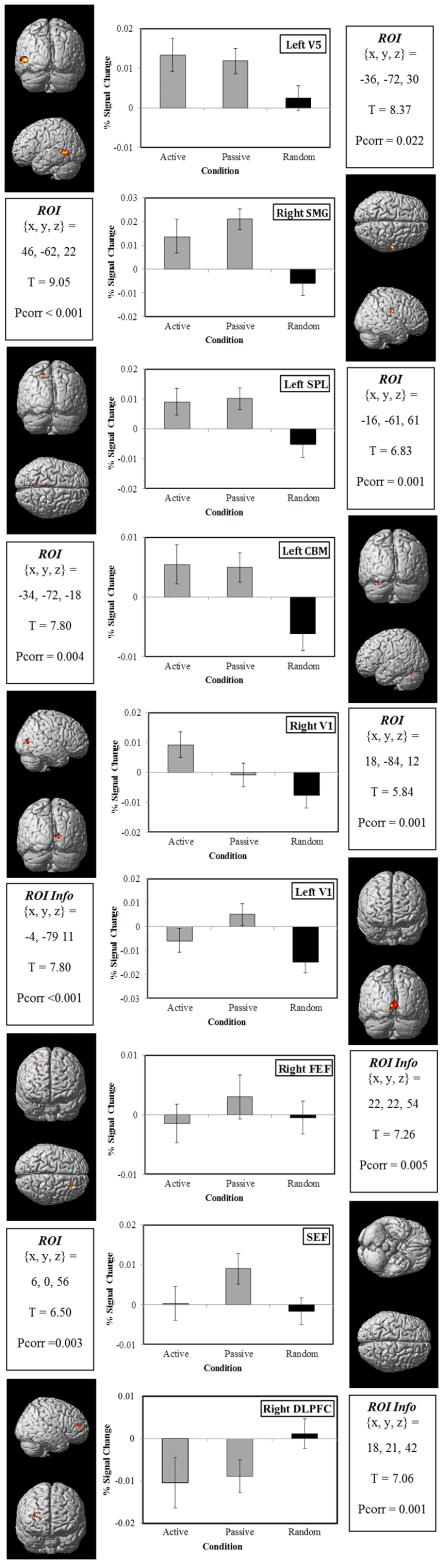
a, b and c: The figures display the mean % BOLD signal change for each of the 3 tasks (Active, Passive and Random) for each delay (2, 4 and 6 s) in the ROI. Figure 3a: shows the medial temporal cortex (V5), the supramarginal gyrus (SMG), and superior parietal lobe (SPL); Figure 3b: presents data for the cerebellum (CBM), and primary visual cortex (V1); Figure 3c: shows data for frontal eye fields (FEF), supplementary eye fields (mid SEF), and dorsolateral prefrontal cortex (DLPFC). The centre of mass for the regions of interest used in the analysis are highlighted within a template brain on either side of the % signal change graph for all figures and brain coordinates are presented using the Montreal Neurological Institute (MNI). T values and significance corrected cluster level P values are also displayed for each ROI.

We used ‘MarsBar’ (http://marsbar.sourceforge.net/ see [23]), an analysis toolbox designed for use with SPM to generate regions of interest (ROI) for the group level activations. We used main effect data (and coordinates) from a previous fMRI experiment [15] to generate ROIs in order to avoid a bias to any single task of interest used in the current study. Furthermore, a priori data from previous studies for areas with activity during a delay also informed this choice. These priori areas involved in memory included: early visual areas (V1) [24], [25], V5 [26], FEF and SEF [9], [27], the DLFPC [4], [15], the superior parietal lobe (SPL) [28], the supramarginal gyrus (SMG) [29], [30], [15] and the cerebellum (CBM) [15]. An 8 mm sphere was positioned around the centre of mass for each of the identified ROIs with significant activations (T>4.5, voxel size >15, FWE of p<0.05) within each subject. The centre of mass is highlighted in figure 3a, b and c for each ROI and a small volume correction was applied to these areas (ROI  = 8 mm) on the maximum activated cluster (Pcorr shown in Figure 3). In this way we have used ROIs for small volume corrections with voxel-wise statistics to avoid type 1 errors. The ROIs comprised a range of bilaterally activated regions including: V1, FEF, SEF, PFC, SPL, and the Cerebellum which are based on brain areas identified in a previous paper [15]. Using these ROIs a standard GLM-based approach was used in which a regression model was solved at each voxel (mass univariate) within the ROI to assess whether signal intensity for each of the delay regressors (2a2, 2a4, 2a6, 2p2, 2p4, 2p6, 2r2, 2r4 and 2r6), for each subject significantly differed from baseline (control task). This data was extracted in order to establish signal level differences in different areas for the different tasks and was subsequently averaged across all subjects (as presented in figure 3).

Statistical tests were used to establish significant differences between the % BOLD signal change for each brain area and task (data shown in figure 3). A single repeated-measure multivariate ANOVA (SPSS, IBM) across all brain areas was used to minimise type 1 errors. This ANOVA had 3 levels: (i) brain area (left V5, right SMG, left SPL, left CBM, left/right BA18/19, right FEF, SEF, right DLPFC), (ii) task (Active, Passive and Random) and (iii) delay (2, 4 and 6 s). All data was checked for sphericity and multivariate tests with Bonferroni post-hoc analysis are reported. Further analysis using the 4 and 6 second delay and the 6 second only across all the brain regions was used for further validation of the differences in memory versus randomized tasks as these delays are thought to be absent of stimulus and motor related activity.

## Results

### Eye Data: Single Subject

Data from a single subject to each of the 3 main tasks are shown below with eye position, eye velocity and target position all plotted against time. The delay between each presentation of the stimulus can also be seen, with the 2 second delay plotted in the upper graph, the 4 second delay in the middle graph, and the 6 second delay on the lower graph.

### Eye Data: Group

The mean eye data from all subjects for this experiment and details of the data analysis and recording has been reported previously [21]. We find clearly anticipatory responses during the Active and Passive tasks to the second presentation of the stimulus in both V50 (velocity 50 ms after target onset i.e. prior to visual feedback) and latency (mean latency: Active  = −260 ms, Passive  = −220 ms; NB negative values denote eye movements prior to target onset) (see Figure 2), but found no anticipation during the random task as expected (mean latency  = 94 ms). We also find a significant non-linear optimization of short-term memory after the 4 seconds delay in V50 to the 2^nd^ stimulus presentation in the predictable responses (p<0.05), however no significant difference between delays was observed in latency (although a trend for earlier onset for the 4 second delay was observed p = 0.075). The random task revealed no anticipation and no difference for any of the delays used (see Figure 2).

### Group fMRI Data – % Signal Change

The group level activations revealed areas well known in generating pursuit eye movements including DLPFC, FEF, SMG and SPL (for details see [22]). Several of these areas were identified as regions of interest (ROI) using the group level activations for all tasks (see figure 3) relative to the control (baseline). We generated ROIs by using these activations, plus a priori knowledge from a directly relevant previous study as mentioned above. We generated 8 mm^3^ spheres encompassing the activation sites of interest and in this way the regions of activation were not biased towards any of our tasks of interest (Active or Passive). We identified 9 areas using this technique that included: Left V5, Right SMG, Right SPL, Left CBM, Left and Right V1, Right FEF, Mid SEF, Mid DLPFC. It is worth noting that the signal level changes are small due to the rapid event-related design of the experiment [31] and because the signal relates to memory processing as opposed to a more robust visual or motor related activity. To identify significant differences we used a repeated measures ANOVA on the mean % signal change responses of each subject for all brain areas to the 3 tasks (Active, Passive and Random) and the 3 delays (2, 4 and 6s). Using this technique we found a number of significant findings. We found an overall weak significant difference in the brain AREAs investigated (F_(2,9)_ = 2.105, p<0.05, η^2^ = 0.174), but the effect of the Task was strong (F_(2,9)_ = 11.63, p<0.001, η^2^ = 0.538). We also obtained an Area * Task interaction (F_(2,9)_ = 3.96, p<0.001, η^2^ = 0.238) and a Bonferroni post-hoc analysis of the Area * Task interaction for all delays is shown in Table 1. For validation purposes a multivariate comparison of the 4 and 6 second delay (excluding the 2 second delay) and the 6 second delay only was performed and a clear and robust Task related effect was observed (4 and 6 delay: F_(2,9)_ = 29.27, p<0.001, η^2^ = 0.867; 6 delay: F_(2,9)_ = 19.02, p = 0.001, η^2^ = 0.809).

**Table 1:** Bonferroni post-hoc analysis of the Area * Task interactions with reported p values.

Area	Task Comparison	P Value
**Left V5**	Random v Active	0.025
	Random v Passive	0.033
**Right SMG**	Random v Active	<0.001
	Random v Passive	<0.001
**Left SPL**	Random v Active	0.013
	Random v Passive	0.008
**Left CBM**	Random v Active	0.020
	Random v Passive	0.004
**Right BA 18/19**	Random v Active	0.03
	Random v Passive	No effect
**Left BA18/19, Right FEF, SEF, and DLPFC**	All	No effect

Post-hoc (Bonferroni) analysis of the Area*Task interaction observed with the GLM repeated-measures ANOVA. The significant P values are shown in the right-hand column, the task comparison in the idle column and the brain area in the left-hand column.

## Discussion

To investigate the storage of motion information needed to generate anticipatory pursuit eye movements in more detail we incorporated a 2, 4 or 6 second delay between stimulus encoding and the anticipatory motor response. Such a delay is a common feature of memory-guided saccade tasks, and has been used extensively to investigate the areas involved in spatial short-term memory during saccades (see [32]). However, this is the first fMRI experimental design to investigate a delay between two presentations of a pursuit stimulus in order to discover the locus of visual motion memory in preparation for an eye movement. The contrast between eye fixation during the delay in the predictive (Active and Passive) tasks and the Random task provides a unique comparison, establishing areas involved in velocity memory, without the confounds of either an eye movement or indeed visually driven response information. It is important to note that during the 2 second delay (and possibly the 4 second) we could have possible contamination from stimulus and motor related activity in the brain from the 1^st^ presentation of the stimulus due to the slowly changing nature of the hemodynamic response. However, we have taken steps in the experimental design and analysis to allow reliable interpretation of the data. First, we have removed stimulus-related effects by contrasting all test tasks (Active, Passive and Random) with a control task that mimicked the stimulus onset, offsets and delays, but without target motion and accompanying eye movement. Secondly, by comparing the Active and Random tasks with the Passive task, we have been able to isolate the motor and stimulus related activity present in all trials during the 1^st^ presentation of the stimulus from non-motor related activity. Using this design, and by comparing Predictable (Active and Passive) and Random tasks we have been able to isolate activity specifically associated with motion memory during the delay. In our analysis we have looked only at the last 2 seconds for each delay to maintain compatibility between delays; however, we are aware that the 4 and 6 second delay provide the strongest evidence for motor storage without any possible stimulus and motor contamination. Because of this we have done a further analysis comparing the 6 second delay in isolation across the different tasks and brain areas. Our results show differences within this 6 second delay for the memory versus randomized tasks, but no differences between the passive versus active acquisition of the information. The discussion will identify each ROI before discussing possible roles of these brain areas during a delay:

### Middle Temporal Area (V5 complex)

This area (including left V5 and right SMG in our study) has a long and established role in motion perception as shown by Maunsell & Van Essen [33] in the macaque; however its role in visual motion memory is much more debatable. Several recent studies into the role of V5 during visual short-term memory for motion include psychophysical evidence, TMS and fMRI. Ong, Hooshvar, Zhang, & Bisley [34] found spatiotopic overlapping of sample and test in a delayed match-test-to-sample task optimises memory performance, with further testing revealing that the critical spatial separation for this performance was equivalent to the receptive fields of neurons in V5. Further evidence of the involvement of V5 in perceptual memory and priming has been demonstrated with TMS [35], [36]. A recent TMS study in which Silvanto and Cattaneo [37] induced a phosphene during visual motion maintenance, found that when the phospene and motion memory spatially overlapped, the phosphene contained features of the motion memory, which did not occur in non-overlapping phosphenes. Further evidence of V5 involvement in visual memory comes from electrophysiological studies on primates [38], [39]. These previous studies all indicate that neurons in V5+ reflect motion information held in visual short-term memory. Alongside this, Bisley and colleagues [39] found the activity in neurons in MT that revealed neural firing signals during a delay up to 3000 ms in primates that was unrelated to priming signals. However, more recent work from this group suggests the activity in MT plays a role in a more sensory comparison during a discrimination task [40].

These earlier findings are consistent with our observations that both left and right V5 and SMG show memory related activity during the 6 second delay in both the Predictable tasks (Active and Passive) for pursuit eye movements, but not during the Random task. The similar findings during the 2 and 4 second delays provide additional support, with the caveat that there may be some persistent influence of the initial presentation. The fact that similar sustained activity was observed during both the Active and Passive predictable tasks suggests that the signal represents memory for the motion of the target and not motor related activity.

Several previous fMRI studies have found a positive or negative correlation of activity in V5 during predictive smooth pursuit in the absence of vision compared with visual tracking, depending on the task [41], [15]. However, these previous fMRI studies have not been designed to look at the maintenance of the signal over several seconds without confounding the signal with the eye movement response. Summarizing this previous information, it seems that V5 plays an important role in processing and maintenance of motion information but is not involved in generating the motor response. These findings appear initially to be in contrast to a recent study in monkeys by Kurkin, Akao, Shichinohe, Fukushima, & Fukushima [42] who used a similar paradigm to the one presented here. Their design also implemented a Go/NoGo task with ∼4 s delays between target presentations and action. They found no sustained activity in dorsolateral MST (MSTd) during the delay. One difference between the methods is that the monkeys obtained global motion information during encoding while fixating (our subjects either followed or didn't follow a single moving target). This initial step was more likely to activate MSTd in Kurkin et al's [42] experiment whereas ventrolateral MST (MSTl) is more likely to be excited by the small targets that we used [43]–[46]. In the Kurkin et al [42] experiment the monkeys eventually chose which one of two oppositely-directed small targets to pursue, based on the direction of the initial global motion stimulus. Since this evoked a reactive, not anticipatory, pursuit response and the target always had the same speed, it is possible that the monkeys were not holding velocity information) but direction information, as, indeed, the authors intended. In contrast, our subjects were required to hold velocity information in order to achieve the observed scaling of the anticipatory responses to the two target velocity levels as shown by the behavioural results [21]. Taken together these findings suggest that maintenance of information in V5 may be specific for speed information rather than direction of motion.

Moreover, maintenance of this activity was similar irrespective of prior motor activity, which is consistent with the evidence that MST is a site where reconstruction of target motion information takes place [45], [42], [27]. Motion memory requirements in our task are similar to motion perception tasks in which current and prior motion stimuli are compared. Using such a task, Greenlee, Lang and Seeger [47] found that patients with superior temporal lobe damage (corresponding to SMG) had higher velocity discrimination thresholds than normals and that thresholds increased as delay between presentations increased from 1 to 10 seconds, supporting the idea that SMG may be critical for sustaining motion memory.

### Superior Parietal Lobe (SPL)

We found some maintenance of activity in the posterior parietal cortex during both the Active and Passive tasks. This activity was not observed in the random task and was also prominent for the 6 second delay in the predictive tasks and hence implies that this area, like V5 and SMG, is also involved in visual short-term memory for motion. The SPL is part of the PPC, with the latter having a well-established role in spatial memory as indicated by TMS, imaging and electrophysiological studies ([48], [32], [49] and for review see [50]). Furthermore, studies have indicated that this visual short-term memory in PPC has a limited capacity [51], is dependent on attentional demands of the task [52], and is important in spatial learning [53]. Our study provides evidence for a role of the SPL in the circuitry involved in motion information storage during a delay providing further evidence of overlapping networks for saccades and smooth pursuit in short-term memory maintenance. It is well established that PPC plays a role in the enhancement of activity in MST/V5 when a target is selected for pursuit [44], [54]. It is possible therefore, that SPL is also responsible for maintaining activity in V5 when similar motion is expected in the second presentation. Lencer et al [14] found a laterality effect in SPL for predictive pursuit, in support of the data presented here, which provides further evidence of velocity storage during a delay being specific to left SPL as also identified in this study. We suspect the sustained activity during the delay in this area, like V5, is not simply related to the after-effects of the 1^st^ presentation of the stimulus, since no sustained activity was observed in the Random task even though this evoked an equivalent eye movement response.

### Cerebellum

The Cerebellum has a well-established role in the generation of eye movements (for review see [55]). Furthermore, the cerebellum has previously been implicated in predictive smooth pursuit, showing a learning related signal during repeated presentations of the same smoothly moving stimulus [15]. The current experiment looks explicitly at the delay between repeated presentations of a stimulus to establish the role of the cerebellum in the maintenance of the signal. The data shows the cerebellum is involved in the maintenance of information needed to generate and anticipatory pursuit eye movement. Interestingly, it may also play different roles depending on the task (predictable versus random). It seems that this area may be involved with either maintaining the Active motor plan or in the development of a generating motor plan in the case of the Passive task. The motor plan would not be necessary in the random task since the velocity is unknown and hence the brain activity in CBM reflects this degradation of activity in randomized tasks. An alternative interpretation is that this area is important in post-encoding during the maintenance of motion information, which it may transmit into longer term memory storage.

### Primary Visual Cortex (V1)

The right hemisphere of V1/V2 seemed to produce a more memory related response to the active motor movement than the random task. The primary visual cortex is principally thought to be a direct retinotopic mapping of the visual field and to be sensitive to both static and moving objects. We suspect the activity of this area during the delay may be related to the difference in the visual representation of the stimulus during the 1^st^ presentation of the stimulus. In the case of the Active task some visual adaptation or priming (short-term storage) may exist that has been found in both early visual cortex [56] and more frontal regions [57]. The reduced signal change in the Random task may suggest the stimulus is not optimally located on the fovea during the Random eye movement task, when compared with fixation, and thus the latter provides greater stimulation.

### Frontal Eye Fields (FEF)

The frontal eye field involvement in the preparation of saccades and generation of smooth eye movements has been established for many years ([58], [59] for a review see [5]). A study by Gaymard et al., [55] revealed the FEF to be involved in short-term memory during memory-guided saccades in a patient with a localized lesion in left FEF. More recently Ding et al [16] and Burke & Barnes [15] found the activity in FEF to be more prominent during visually-guided pursuit and specifically associated with the premotor drive. In contrast, a recent paper by Fukushima et al [60] revealed neurons in the FEF to be active in response to the predictive component of the movement, but not during a delay in the Active/Passive task. Our results reveal that there is no difference between any tasks for this brain area implying that the FEF is not specifically important in holding predictive pursuit information.

### Supplementary Eye Fields (SEF)

Much research progress has been made recently into the involvement of the SEF during predictive pursuit eye movements [9], [61], [62]. These previous studies have shown either facilitation of predictive pursuit during stimulation [62] or continued single unit activity during a delay in anticipation [9]. In contrast, our results show a similar effect as in FEF, and no delay related activity was observed in this area during the anticipatory tasks in this study.

### Dorsolateral Prefrontal Cortex (DLPFC)

The final region of interest in our investigation was dorsolateral prefrontal cortex. This area has also been implicated in the storage/modulation/decisional role of velocity storage information during predictive smooth pursuit [50], [41], [15], [16]. The results presented here suggested the DLPFC is not explicitly involved in the storage of motion information during smooth pursuit, consistent with previous studies that have suggesting a role for this area in the selection of an appropriate motor response.

## Conclusions

**Figure 4:**
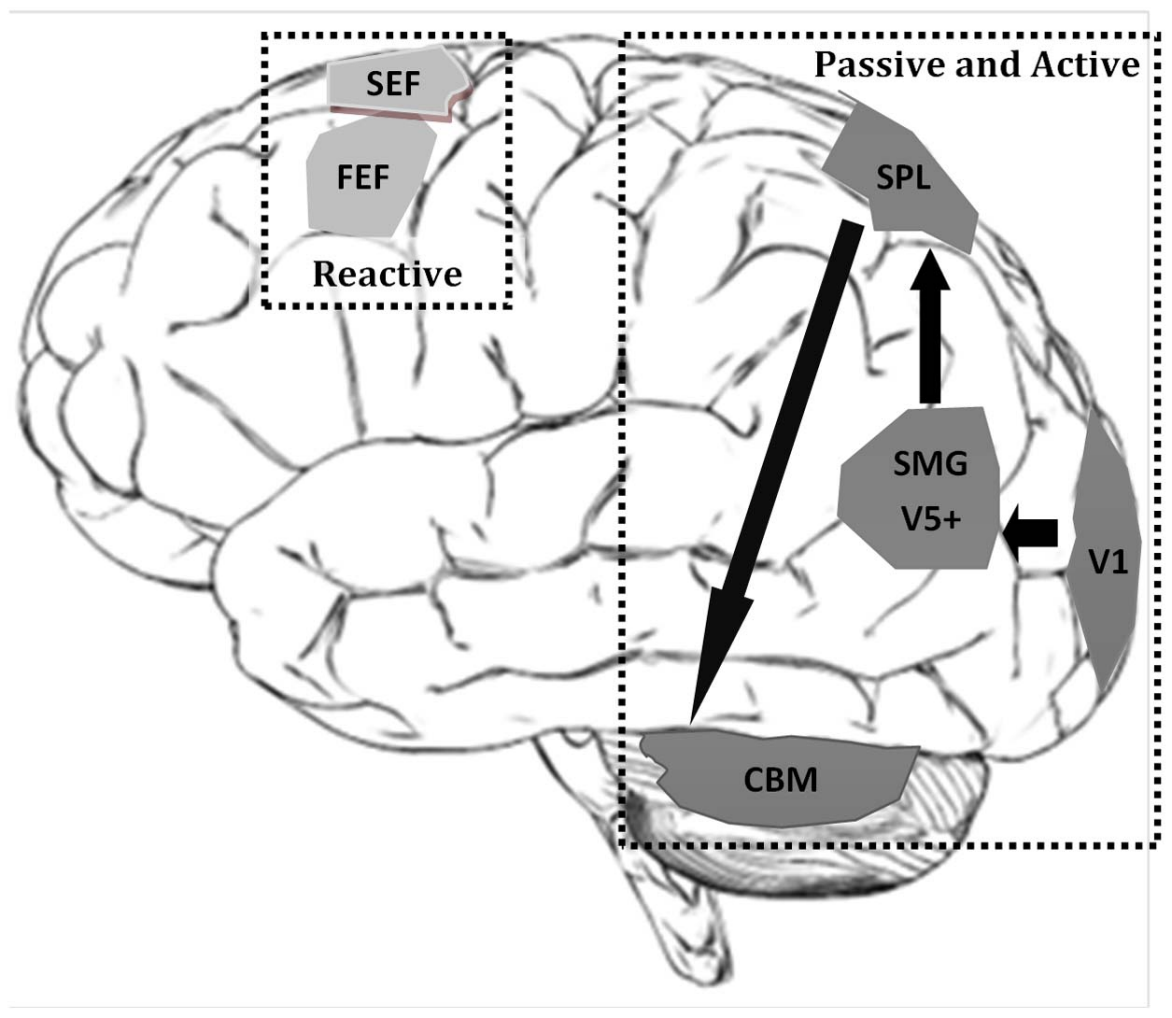
A summary diagram to show the areas involved in short-term memory maintenance during predictive smooth pursuit eye movements. Light grey areas are areas more involved in the reactive tasks, and the darker grey areas are important in both Active and Passive predictable tasks.

The concept of memory for motion is familiar in the context of a sustained pursuit response during brief target disappearance [63], [64] and the associated maintenance of sustained activity in area MST [3]. However, as shown previously [20], [65] and reinforced by the current behavioural evidence, memory for motion can be much more complex. Our observation of sustained activity during delays of 2–6 s in SMG is not unexpected given the already established role of MST in reconstruction of target motion. Given the similar sustained activity in SPL, an area classically associated with attention, our suggestion is that left SPL and SMG form part of a positive feedback loop that is responsible for sustaining the activity in SMG in the manner depicted in Fig 4. The activity observed in the cerebellum may also indicate that it forms part of this network.
