# Correction: In Pursuit of Delay-Related Brain Activity for Anticipatory Eye Movements

**DOI:** 10.1371/annotation/f59e8c63-4f95-47f6-8d90-00348f4a52e4

**Published:** 2013-12-19

**Authors:** Melanie R. Burke, Graham R. Barnes

There is an error in the legend for Figure 3. The legend refers to figures 3a, 3b and 3c, which is not correct. Figure legend 3 should read:

Figure 3: The figures display the mean % BOLD signal change for each of the 3 tasks (Active, Passive and Random) for each delay (2, 4 and 6s) in the ROI. The graphs show: the medial temporal cortex (V5), the supramarginal gyrus (SMG), and superior parietal lobe (SPL), the cerebellum (CBM), primary visual cortex (V1), the frontal eye fields (FEF), supplementary eye fields (mid SEF), and dorsolateral prefrontal cortex (DLPFC). The centre of mass for the regions of interest used in the analysis are highlighted within a template brain on either side of the % signal change graph for all figures and brain coordinates are presented using the Montreal Neurological Institute (MNI). T values and significance corrected cluster level P values are also displayed for each ROI. 

